# Enhanced Enzymatic Hydrolysis and Structural Features of Corn Stover by NaOH and Ozone Combined Pretreatment

**DOI:** 10.3390/molecules23061300

**Published:** 2018-05-29

**Authors:** Wenhui Wang, Chunyan Zhang, Shisheng Tong, Zhongyi Cui, Ping Liu

**Affiliations:** 1 College of Food Science and Nutritional Engineering, China Agricultural University, Beijing 100083, China; wwhui123321@163.com (W.W.); m18612203994@163.com (C.Z.); zhongyi.cui@dupont.com (Z.C.); 2 Bio-Pharmaceutical College, Beijing City University, Beijing 100094, China; shishengt@163.com

**Keywords:** corn stover, alkali, ozone, combined pretreatment, enzymatic hydrolysis, surface morphology, structural characteristics

## Abstract

A two-step pretreatment using NaOH and ozone was performed to improve the enzymatic hydrolysis, compositions and structural characteristics of corn stover. Comparison between the unpretreated and pretreated corn stover was also made to illustrate the mechanism of the combined pretreatment. A pretreatment with 2% (*w*/*w*) NaOH at 80 °C for 2 h followed by ozone treatment for 25 min with an initial pH 9 was found to be the optimal procedure and the maximum efficiency (91.73%) of cellulose enzymatic hydrolysis was achieved. Furthermore, microscopic observation of changes in the surface structure of the samples showed that holes were formed and lignin and hemicellulose were partially dissolved and removed. X-ray Diffraction (XRD), Fourier Transform Infrared Spectroscopy (FTIR) and Cross-Polarization Magic Angle Spinning Carbon-13 Nuclear Magnetic Resonance (CP/MAS ^13^C-NMR) were also used to characterize the chemical structural changes after the combined pretreatment. The results were as follows: part of the cellulose I structure was destroyed and then reformed into cellulose III, the cellulose crystal indices were also changed; a wider space between the crystal layer was observed; disruption of hydrogen bonds in cellulose and disruption of ester bonds in hemicellulose; cleavage of bonds linkage in lignin-carbohydrate complexes; removal of methoxy in lignin and hemicellulose. As a result, all these changes effectively reduced recalcitrance of corn stover and promoted subsequent enzymatic hydrolysis of cellulose.

## 1. Introduction

In an effort to reduce the energy crisis and the environmental pollution, preparation of recycled lignocellulosic biomass for the use of energy, materials and chemicals has become the focus of today’s research. Due to the low degree of lignification, high carbohydrate content and easy absorption of carbohydrate, corn stover has high value in comparison with other lignocellulosic biomass [1]. Pretreatments with physical, chemical and biological methods, however, are necessary to change complex network structure among cellulose, hemicellulose and lignin in corn stover, ascertain pretreatment can release the closure and reduce the strong interchain between lignin and cellulose, compromise the crystalline structure of cellulose, enhance accessibility of enzyme and make corn stover fully utilized [2]. With existing pretreatment methods, sodium hydroxide can rupture the interchain between lignin and other carbohydrates significantly, saponify the inter-molecular ester bonds between hemicellulose and other components, make lignocellulosic swell to remove lignin effectively [3,4]. In addition, this pretreatment not only increases the porosity and internal specific surface area of fibrous materials to ensure effective contact of the enzyme with fibrous materials and degrade it but also changes the structure of lignocellulose and improves its digestibility of polysaccharide by increasing cellulose conversion rate [5]. Because of strong oxidation, low solubility and selective oxidation of ozone, ozone oxidation technology has some limitations in gas-liquid transfer such as the slow rate, high cost and low ozone utilization rate, which makes it difficult to be used alone [6,7]. Ben’koet et al. used ozone to pretreat aspen wood and found that the efficiency of enzymatic hydrolysis was determined by the absorption rate of ozone [8]. Panneerselvam et al. used different ozone concentrations of 40 mg/L, 50 mg/L, 58 mg/L to treat energy grass [9]. Pretreatment conditions and results showed that ozone treatment can remove lignin effectively without cellulose degradation. Bule et al. used ozone to pretreat wheat stover, the particle size of which was less than 60 mesh, for 2 h and the results showed that the lignin structure was modified significantly and the sugar recovery rate increased from 13.11% to 63.17% in comparison with untreated samples [10]. The previous experiment made corn stover treated in 2% NaOH solution at a temperature of 80 °C for 2 h. The specific surface area diameter of corn stover particles was reduced from 189.9 μm to 132.2 μm, and the specific surface area of stover decreased after ozone treatment at pH 5 for 50 min up to 93.11 μm, compared with the specific surface area diameter of the non-alkaline control group decreased by 51%, indicating that alkali combined with ozone made the stover particles smaller by removing lignin. This result was shown in Supplementary Materials.

In this work, a two-step pretreatment using NaOH and ozone was performed on corn stover to improve its enzymatic hydrolysis and changes in compositions and structural characteristics compared to unpretreated. Pretreated corn stover was also analyzed to illustrate the mechanism of the combined pretreatment.

## 2. Materials and Methods

### 2.1. Materials and Sample Preparation

The corn stover was obtained from the farm research fields at the Jilin Agricultural University (Changchun, Jilin, China). After the corn stover sample was cut into small pieces, it was oven-dried to bring down the moisture content, then milled and screened to particle size of less than 1 mm. The dry sample was kept at −20 °C for future use.

### 2.2. Methods

#### 2.2.1. NaOH Treatment

Two gram of dry corn stover and 30 mL of 2% (*w*/*v*) NaOH were mixed completely in a 50 mL centrifugal tube reactor, which was then incubated in a water bath for 2 h–4 h at 40 °C, 60 °C and 80 °C. The pretreatment conditions including NaOH treatment temperatures and times, shown in Table 1. When the reaction was over, the tube reactor was cooled to room temperature and filtered via a 300-mesh sieve to separate the mixture into the solid residue and liquid hydrolysate. The solid residue was rinsed with deionized water or saturated carbon dioxide water until it reached neutral pH.

#### 2.2.2. Ozone Treatment

Two gram of sample and 30 mL deionized water were placed in a 60 mL of beaker to prepare for ozone pretreatment. 2 mol/L of dilute sulphuric acid was also added to adjust the initial pH of the reaction liquid. Ozone was generated by an ozone generator (CF-10F, Beijing, China). During the reaction, ozone concentration maintained at 78 mg/L for different time with magnetic stirring (85-2, Shanghai, China) at a room temperature. Ozonation experimental conditions are shown in Table 2.

#### 2.2.3. Combined Sodium Hydroxide and Ozone Pretreatment

The experiment is divided into two processes: NaOH Pretreatment and Ozone Pretreatment. The experimental design of the alkali treatment stage is a two-factor three-level, as shown in Table 1. Each group of experiments after alkali treatment was further treated with ozone. The experimental design of the ozone treatment stage is a two-factor two-level, as shown in Table 2. Combined pretreatment samples were prepared for enzymatic hydrolysis and other analysis. The pretreatment conditions, including NaOH and ozone treatment, were optimized for high delignification and high cellulose composition. For convenient description of structure characterization, the special nomenclature for combined pre-treatment conditions were as follows: A-5-25: 2% NaOH at 80 °C for 2 h and the ozone initial pH 5 for 25 min. A-9-25: 2% NaOH at 80 °C for 2 h and the ozone initial pH 9 for 25 min. A-9-35: 2% NaOH at 80 °C for 2 h and the ozone initial pH 9 for 35 min. B-9-25: 2% NaOH at 80 °C for 3 h and the ozone initial pH 9 for 25 min. C-9-35: 2% NaOH at 60 °C for 2 h and the ozone initial pH 9 for 35 min. D-9-35: 2% NaOH at 80 °C for 4 h and the ozone initial pH 9 for 35 min. Blank: untreated degreased stover.

#### 2.2.4. Enzymatic Hydrolysis

Pretreated stover samples, of 0.2 g in 100 mL, were placed in an Erlenmeyer flask and added to 10 mL acetate buffer (0.1 mol/L, pH 4.8), which was prepared with sterile water and contained 40 μg/mL tetracycline, 30 μg/mL cycloheximide and 40 μL xylanase solution. The mixture was incubated in shaking bath (120 rpm) at 70 °C for 24 h. After the reaction, the sample was cooled down to room temperature, 40 μL cellulase and 30 μL β-glucosidase were added and it was then incubated at 50 °C for 72 h. Cycloheximide could inhibit the DNA translation of eukaryotes to stop cell growth or even cause death. The purpose of adding cycloheximide and tetracycline hydrochloride was to inhibit the growth of microorganism which influenced the pH value during the enzymatic process and affected enzyme activity. Enzymatic hydrolysate was filtered through 0.22 μm membrane and then analyzed by HPLC to determine the glucose content and calculated the cellulase hydrolysis rate.

#### 2.2.5. Determination of the Composition Content of the Corn Stover Samples

In this paper, three components (cellulose, hemicellulose and lignin) in the stover before and after pretreatment were determined by two-step acid hydrolysis method (NREL, 2008b). The content of cellulose and hemicellulose, the lignin removal rate was determined by the following equation:

(1) $$\mathrm{Cellulose}\;\mathrm{concent}\;\left(\%\right)\;=\;\frac{C_{1}\times V\times 0.9\;}{m}\;\times 100,$$

(2) $$\mathrm{Hemicellulose}\;\mathrm{content}\;\left(\%\right)\;=\;\frac{C_{2}\times V\times 0.88\;}{m}\;\times 100,$$

In the equation, C_1_ was the concentration of glucose measured by HPLC (mg/mL); C_2_ was the concentration of xylose measured by HPLC (mg/mL); V was the total volume of the reaction system (87 mL); m was the dry weight of the sample (300 mg); 0.9 was the conversion of glucose to cellulose, 0.88 was the conversion of xylose to hemicellulose.

(3) $$W_{1}\;\left(\%\right)\;=\;\frac{m_{1}-{\;m}_{2}\;}{0.3}\;\times 100,$$

In the equation, W_1_ was the acid-insoluble lignin in the stover; m_1_ was the total weight of the sand core funnel and the residue; m_2_ was the weight of the sand core funnel; 0.3 was the dry weight of the sample. The unit of measurement was g.

(4) $$W_{2}\;\left(\%\right)\;=\;\frac{{\mathrm{OD}}_{320}\times \;V\;\times n\;}{300}\;\times 100,$$

In the equation, W_2_ was the acid-soluble lignin in the stover; V was the total volume of the reaction system (87 mL); 300 was the dry weight (units: mg) of the sample; OD_320_ was the absorbance at 320 nm, 30 L/g·cm; n was the dilution factor.

(5) $$W_{3}\;\left(\%\right)\;=\;\frac{m_{4}\;}{m_{3}}\;\times 100,$$

In the equation, W_3_ was the lignin removal rate, the total weight of acid-soluble lignin and the acid-insoluble lignin were the total lignin content of the stover; m_3_ was the total lignin weight of the untreated stover; m_4_ was the total lignin weight of the pretreatment stover.

### 2.3. Structural Analysis

#### 2.3.1. Scanning Electron Microscope (SEM) Analysis

Measured stover samples were placed in an oven for 24 h at 50 °C to remove moisture, imaged with S-3400n scanning electron microscope with a voltage of 20 kV, current of 30 mA and distance of 11.3 mm. Electron microscopy was amplified at different rates to observe the surface morphology of the sample.

#### 2.3.2. X-ray Diffraction (XRD) Analysis

The samples were examined by X-ray diffractometer with CuKa radiation (λ = 0.154 nm). CuKa radiation was eliminated with nickel. The operation voltage and current was 40 kV and 40 mA respectively. The measurement method was θ/2θ linkages scanning. The range of 2θ was 5° to 70°. The step was 0.02° and the time interval was 0.2 s. The sample was pressed at 40 °C and subjected to a 2θ intensity curves. Using Origin and MDI jade 5.0 for data analysis.

#### 2.3.3. Fourier Transform Infrared Spectroscopy (FTIR) Analysis

The samples were placed in an oven at 50 °C for 24 h to remove moisture. 10 mg of dry sample was mixed with 200 mg KBr, manually ground in an agate mortar and pressed at 20 MPa for 2 min in oil pressure. The tablets were placed on a sample rack for FTIR spectra spectroscopy and the spectra was recorded between 4000 and 400 cm^−1^. The PerkinElmer Spectrum and Origin software were used for data analysis.

#### 2.3.4. Cross-Polarization Magic Angle Spinning Carbon-13 Nuclear Magnetic Resonance (CP/MAS ^13^C-NMR) Analysis

Solid-state cross-polarization magic angle spinning was performed on an Agilent 600 M spectrometer operating. The cellulose-rich solid residue sample was packed tightly into the 4 mm ZrO_2_ rotor, 150.81 MHz, spun at 12 kHz at 40 °C. The contact time for cross-polarization was set to 1 ms and delayed for 3 s.

## 3. Results and Discussion

### 3.1. Enzymatic Hydrolysis and Composition of Pretreated Corn Stover

**Figure 1:**
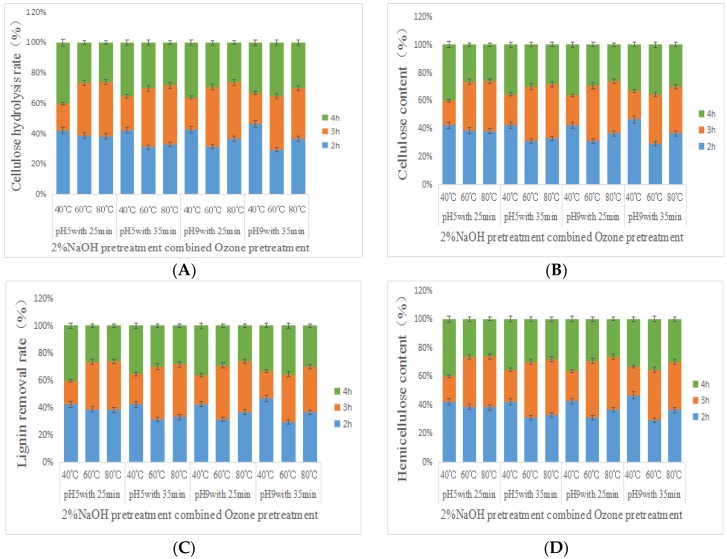
Results of enzymatic hydrolysis and content of cellulose, lignin and hemicellulose in corn stover after NaOH-ozone pretreatment. (**A**) Cellulose hydrolysis rate; (**B**) Cellulose content; (**C**) Lignin removal rate; (**D**) Hemicellulise content.

The stover was co-pretreated by NaOH and ozone and the three compontent content and cellulose enzymolysis were shown in Figure 1. The initial pH at 9 before ozone treatment, which was more conducive to cellulose enzymatic hydrolysis, than pH 5 and the ozone treatment time that conducive to cellulose enzymatic hydrolysis was 25 min > 35 min. When the stover was treated with 2% NaOH at 80 °C for 2 h and ozone treatment condition was the initial pH 5 for 25 min, the maximum enzymatic hydrolysis rate was 86.84%. When the stover was treated with 2% NaOH at 80 °C for 2 h and ozone treatment conditions were the initial pH 9 for 25 min, the maximum enzymatic hydrolysis rate was 91.73%. The effect of the three components in the pretreated stover on the enzymatic hydrolysis of cellulose was different due to the pretreatment conditions. The relative content of cellulose in the stover was 62.48%, the removal rate of lignin was 84.35% and the relative content of hemicellulose was 13.74% after the best pretreatment combination. The correlation between hemicellulose content and cellulose enzymatic hydrolysis was significant (*p* = 0.037 < 0.05). The correlation between hemicellulose content and cellulose enzymatic hydrolysis was significant (*p* = 0.037 < 0.05). The significant difference between the cellulose content and cellulose enzymatic hydrolysis rate was found to be *p* = 0.000 (<0.05) which meant their relevance was extremely significant. The significant difference between the lignin removal and the cellulose enzymolysis rate was found to be *p* = 0.017 (<0.05), indicating that the enzymatic hydrolysis of cellulose was significantly affected by lignin removal.

### 3.2. SEM Analysis

**Figure 2:**
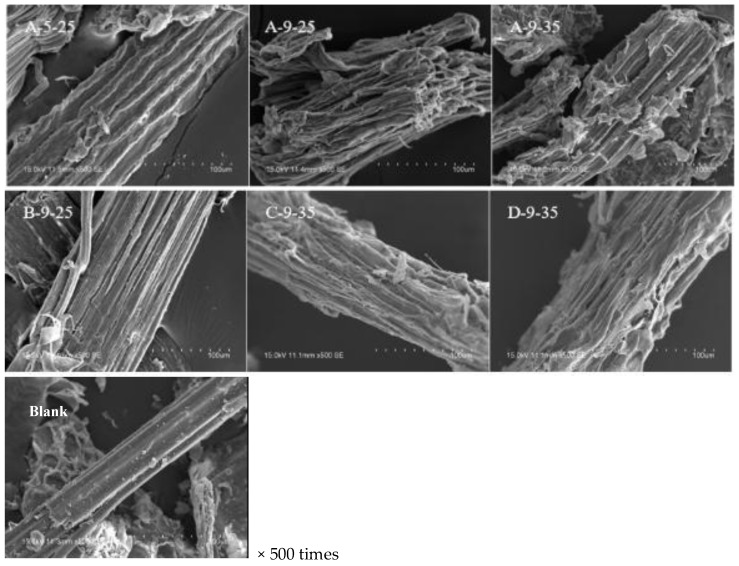
Scanning electron microscopy (SEM) images of samples before/after the combined pretreatment. A-5-25: 2% NaOH at 80 °C for 2 h and the ozone initial pH 5 for 25 min. A-9-25: 2% NaOH at 80 °C for 2 h and the ozone initial pH 9 for 25 min. A-9-35: 2% NaOH at 80 °C for 2 h and the ozone initial pH 9 for 35 min. B-9-25: 2% NaOH at 80 °C for 3 h and the ozone initial pH 9 for 25 min. C-9-35: 2% NaOH at 60 °C for 2 h and the ozone initial pH 9 for 35 min. D-9-35: 2% NaOH at 80 °C for 4 h and the ozone initial pH 9 for 35 min. Blank: untreated degreased stover.

The surface structure of the stover before and after pretreatment is shown in Figure 2. It was found that the surface of untreated (blank) degreased stover was smooth, intact, dense. After the synergistic treatment, change in the surface of the stover was obvious. The density structure was damaged to different degrees, the surface of the stover was fluffy and full of holes, depressions and cracks that increased its specific surface area. In addition, a significant peeling phenomenon appeared on the surface, which indicated that the silica protrusions, waxes and bolts on the outer surface of corn stover were basically cleaned up after synergistic treatment. In A-9-25, we could see fluffy, neat and ordered fiber bundles along the fiber, which indicated that the synergistic treatment could effectively remove ingredients wrapped outside cellulose and break the complex network structure of lignocellulos.

In comparison with A-9-25 and B-9-25, we could see that the stover surface of A-9-25 had more pores, less fiber bundles filler and larger gap, the surface mechanical tissue outside stover was exposed, the cell wall was relaxed, the outer wall specific surface area increased, indicating that 2% NaOH was capable to expand the fiber structure than 4% NaOH. As a result, the enzymatic hydrolysis had better penetration into the cellulose and improved the accessibility of the enzyme [11]. Cellulose content showed that A-9-25 (62.48%) < B-9-25 (69.64%). This angle indicated that the factors affected the contact of cellulose and the enzyme, such as the swelling of the fiber material, the impact of the pores on the cellulose hydrolysis rate was greater than the increase of the fiber content in the stover. Studies have also shown that the enzymatic hydrolysis rate of cellulose and cellulose swelling degree had a linear relationship [12]. In comparison with A-9-25, B-9-25 and A-9-35, it could be roughly concluded that the effect of ozone treatment time on cellulose content and subsequent enzymatic hydrolysis was higher than that of NaOH treatment concentration.

In comparison with A-9-35, C-9-35 fiber bundle surface had a translucent thin layer of material and fluffy scaly structure, the degree of damage was less than A-9-35, indicating that 2% NaOH treatment at 80 °C for 2 h compared to 60 °C treatment 2 h on the stover surface structure damage was greater and it was consistent with the results of low lignin and lower lignin removal rate and lower cellulose enzyme hydrolysis rate (61.45%) in the C-9-35 stover. This may be attributed to the fact that the 80 °C solution allowed the NaOH solution to penetrate better into the cellulose crystallization zone, better weakened the intermolecular or intramolecular hydrogen bonding forces of the cellulose, resulting in better defatting of the degreased stover [13].

The degree of destruction of A-9-35 stover was greater than D-9-35. Combined cellulose content A-9-35 (65.76%) > D-9-35 (58.98%), hemicellulose content A (11.17%) > D-9-35 (9.15%) and lignin removal rate A-9-35 (81.65%) < D-9-35 (88.43%), we could see that pretreatment at a high temperature (80 °C) with a long time could remove more lignin and reduce cellulose and it was consistent with the result that the enzymatic hydrolysis rate of A-9-35 was about 18% higher than that of D-9-35.

In comparison with A-5-25 and A-9-25, surface structural damage degree was A-9-25 > A-5-25. It was consistent with the result of enzymatic hydrolysis A-9-25 > A-5-25, cellulose content A-9-25 > A-5-25, lignin removal rate was A-9-25 > A-5-25. It also showed that the pretreatment effect at the initial pH 9 for 25 min was better than that at pH 5 for 25 min.

### 3.3. XRD Analysis

**Figure 3:**
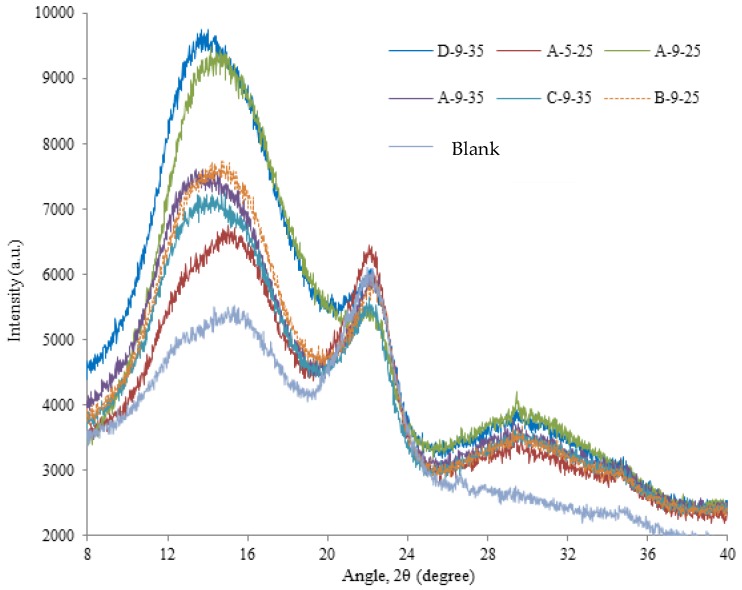
X-ray diffraction (XRD) patterns of the samples before/after combined pretreatment. A-5-25: 2% NaOH at 80 °C for 2 h and the ozone initial pH 5 for 25 min. A-9-25: 2% NaOH at 80 °C for 2 h and the ozone initial pH 9 for 25 min. A-9-35: 2% NaOH at 80 °C for 2 h and the ozone initial pH 9 for 35 min. B-9-25: 2% NaOH at 80 °C for 3 h and the ozone initial pH 9 for 25 min. C-9-35: 2% NaOH at 60 °C for 2 h and the ozone initial pH 9 for 35 min. D-9-35: 2% NaOH at 80 °C for 4 h and the ozone initial pH 9 for 35 min. Blank: untreated degreased stover.

Both the crystalline structure and crystal grain index of cellulose played an important role in the enzymolysis efficiency. In order to study the structural changes of stover cellulose after co-treatment, X-ray diffraction analysis of stover before and after pretreatment was showed in Figure 3.

The peaks at 2θ of 13–17° and 20–23° in Figure 3 exhibited more homogeneous polycrystalline of cellulose [14,15]. All tested samples had significant cellulose surface peaks at 2θ of 15.2° and 22.1°. According to the literature, cellulose I had two crystalline forms, named cellulose Iα and Iβ, different XRD spectra depended on the proportion of these two fiber morphology [16]. After pretreatment, the diffraction peak near 15.2° changed obviously. The diffusion peak of untreated stover shifted to the lower position and the peak shape became high and sharp, which indicating that the spacing of the cellulose microcrystals increased and the stacking density decreased. Specifically, the peak of untreated stover and A-5-25 treatment group were close to 15.2° (100, Iα) but the peak angle after the treatment of B-9-25 and A-9-25 shifted to 14.7° (100, Iα), the peak angle after pretreatment of C-9-35 shifted to 14.1° (10-1, Iα) and the peak angle of A-9-35 and D-9-35 were reduced to 13.8° (011, Iβ). By comparison, NaOH treatment at 80 °C, ozone initial pH 9 for 35 min could reduce the intergranular layer spacing but the ozone initial pH 5 did not have this effect. It also showed that synergistic pretreatment had a great effect on the change of stover crystal grain index. The peak intensity in the 100 crystal plane of A-9-25 was stronger than that of 011 crystals in A-9-35, which may be the key reason for the difference of enzymatic hydrolysis effects.

After pretreatment, the crystal diffraction peak amplitude of 020 near 22.1° at 2θ was small and the peak intensity reduced obviously, indicating that the pretreatment did not have a significant effect on the distance of the crystal layer of the crystal grain. The pretreatment group showed weak peaks near 26.7° (201, Iα), 27.8° (20-1, Iα) and 34.7° (004, Iβ), showing the characteristic structure of natural cellulose I. The stover sample after co-pretreatment of NaOH-ozone, 201 and 20-1 crystal faces disappeared. The new diffraction peak (022) formed at 2θ of 29.5° and proved the presence of cellulose II, which indicated that the stover sample after pretreatment was a mixed crystal structure of cellulose I and II [17]. The change of crystal structure and grain index promoted the hydrolysis of the cellulose and the enzymatic hydrolysis of cellulose occurred more easily in crystal face 100 and newly formed crystal face 022 [18].

D-9-35 had the highest peak intensity at 2θ of 13.8°, mainly due to its high lignin removal rate. Zhao et al. pretreated bagasse with peracetic acid and found that CrI increased because of the removal of lignin [18]. This was consistent with the result of our study that peak intensity of D-9-35 at 2θ = 22.1°, was higher than A-9-35, indicating that 2% NaOH treatment was more conducive than 4% NaOH to the subsequent increase in the rate of enzymatic hydrolysis. The peak intensity of A-9-25 at 2θ = 14.7° and 29.5° was significantly higher than that of B-9-25 and the peak intensity at 2θ = 22.1° was significantly lower than that of B-9-25, indicating that 2% NaOH was more conducive to the hydrolysis than 4% NaOH.

In summary, the peak intensity of A-9-25 near 14.7° and 29.5 at 2θ were much higher than that of other pretreatment groups, the peak intensity of 22.1° at 2θ was the lowest. These results indicated that the microcrystalline structure of stover treated after 2% NaOH at 80 °C for 2 h and the initial pH 9 of ozone for 25 min had shifted, which was conducive to enzymatic hydrolysis.

### 3.4. FTIR Analysis

**Figure 4:**
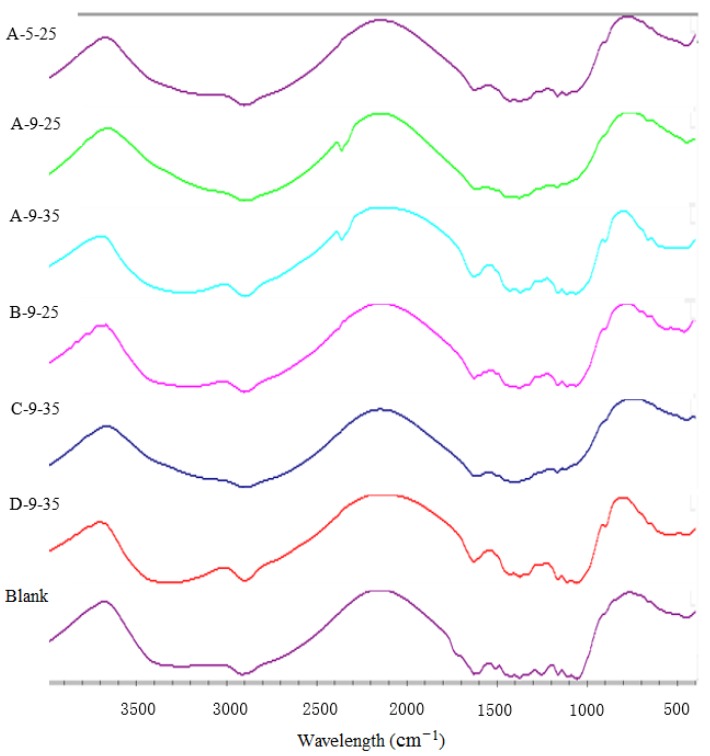
**Fourier transform infrared** (FTIR) spectra of the samples before/after combined pretreatment. A-5-25: 2% NaOH at 80 °C for 2 h and the ozone initial pH 5 for 25 min. A-9-25: 2% NaOH at 80 °C for 2 h and the ozone initial pH 9 for 25 min. A-9-35: 2% NaOH at 80 °C for 2 h and the ozone initial pH 9 for 35 min. B-9-25: 2% NaOH at 80 °C for 3 h and the ozone initial pH 9 for 25 min. C-9-35: 2% NaOH at 60 °C for 2 h and the ozone initial pH 9 for 35 min. D-9-35: 2% NaOH at 80 °C for 4 h and the ozone initial pH 9 for 35 min. Blank: untreated degreased stover.

The characteristic absorption peaks of cellulose, hemicellulose and lignin in infrared spectrum was shown in Table 3. The FTIR spectra of untreated and pretreated stover were measured in Figure 4. The carbonyl at 1737 cm^−1^ was esterified (polyxylose C=O conjugate) and came from the ester bond between the acetyl group attached to xylose and glucuronic acid, the peak was much stronger in spectra of untreated stover than that of the treated. This indicated that co-pretreatment could remove hemicellulose ester linkages [19].

Wavelength 1512 cm^−1^ belonged to the stretching of lignin aromatic ring –C=C– aromatic skeleton. The peak was characteristic in lignin indicating G > S [20]. The sharp band almost disappeared in spectra of treated stover but had stronger absorbance in spectra of the untreated sample. The decrease or disappearance of peak intensity could be attributed to the removal of aromatic ring lignin and the destruction of the lignocellular structure in the residue under the corresponding pretreatment conditions [21]. This was consistent with the chemical composition of the sample. It showed that ozone treatment could reduce the content of –C=C– in wheat stover [10].

Due to the breakage of the bond between the lignin-carbohydrate after pretreatment, the peak at 1250 cm^−1^ was evident in the control group and was weak in the other groups [16]. The peak at 1320 cm^−1^ was much stronger in the spectra of the control group than that of other groups, suggesting that the guaiacyllignin (G) structure of the lignin in the residue was destroyed after pretreatment. Compared to the peak intensities of A-9-25 and A-9-25, it showed that 25 min ozone treatment was more favorable for removing the G structure. According to the literature, the toughness of G structure was higher than that of S structure [22,23], so the destruction of G structure was more conducive to subsequent enzymatic hydrolysis.

### 3.5. ^13^C-NMR Analysis

It could be seen from the figure that most of the signals in the ^13^C-NMR spectrum of the samples after the pretreatment were similar. Compared with the non-pretreated samples, the peaks in the 20–35 ppm region were weakened in the A-5-25 group and almost disappeared in the other groups. The disappearance of the peak in the region or the decrease of the peak intensity showed that stover pretreated only trace amounts of residual lignin. It was also noted that the initial treatment with ozone at pH 9 from the initial ozone at pH 5 removed the lignin more. The result was consistent with the 65.40% removal rate of lignin treated in A-5-25 in chapter 3. Compared A-9-25 and A-9-35, C_1–6_ signal strength corresponding to the former peak to the latter were significantly stronger and sharper peak shape, indicating that the pretreatment of ozone for 25 min compared to 35 min, the greater degree of damage to the stover, the cellulose could be better separated, the higher relative content of cellulose was more conducive to subsequent enzymatic hydrolysis.

Compared A-9-35 and D-9-35, no new peak appeared and no old peak disappeared, indicating that the type of carbon in the carbohydrate compound did not change when the treatment time of 2% NaOH at 80 °C increased from 2 h to 4 h. D-9-35 compared with the non-pretreatment group, the carbon signal peak was sharper, indicating that the treatment of 2% NaOH at 80 °C for 4 h with the ozone initial pH 9 for 35 min resulted in the high purity separation of the components in the stover.

The peaks in the 106–153 ppm region were significantly higher in A-9-25 than in B-9-25 and the peaks in B-9-25 almost disappeared, indicating that 2% NaOH at 80 °C for 2 h had less lignin removal and lower cellulose relative content than 4% NaOH. Compared with the above conclusions, the effect of NaOH concentration on composition of stover was greater than that of NaOH treatment time. Compared B-9-25 and untreated group, the peak intensity of 4% NaOH treatment was higher and sharper than that of untreated group, suggesting that 4% NaOH at 80 °C for 2 h with ozone initial pH 9 for 25 min made stover component separated in high purity. The spectra of A-9-35 and C-9-35 were similar and there was no change in peak number and intensity. For ^13^C-NMR, it was impossible to determine the difference between NaOH treatment temperatures 80 °C and 60 °C.

**Figure 5:**
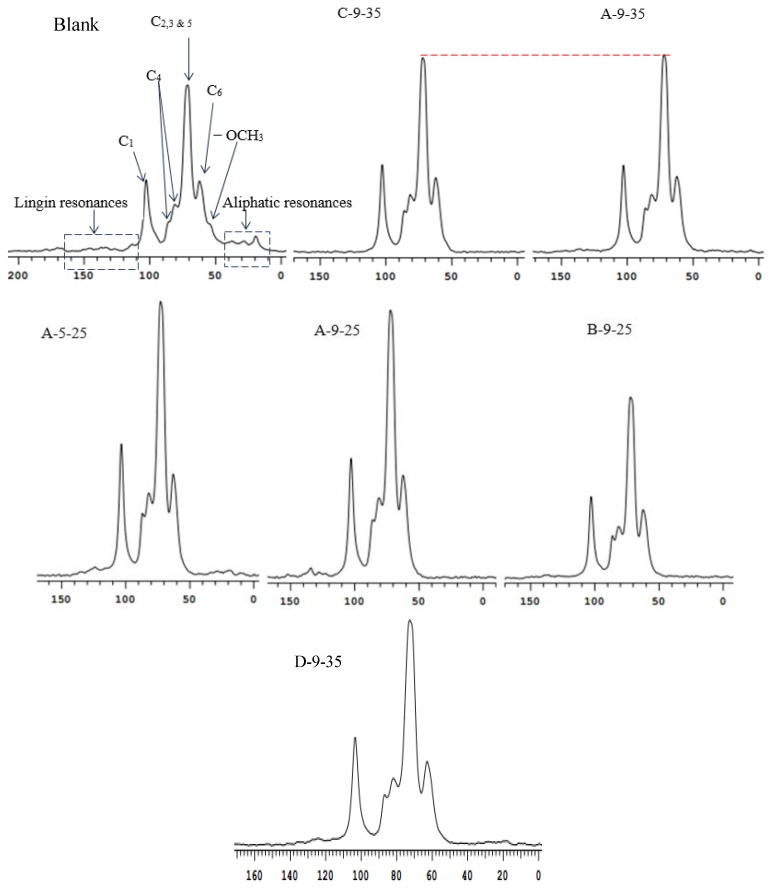
Cross-Polarization Magic Angle Spinning Carbon-13 Nuclear Magnetic Resonance (CP/MAS ^13^C-NMR) spectra of the samples before/after combined pretreatment. A-5-25: 2% NaOH at 80 °C for 2 h and the ozone initial pH 5 for 25 min. A-9-25: 2% NaOH at 80 °C for 2 h and the ozone initial pH 9 for 25 min. A-9-35: 2% NaOH at 80 °C for 2 h and the ozone initial pH 9 for 35 min. B-9-25: 2% NaOH at 80 °C for 3 h and the ozone initial pH 9 for 25 min. C-9-35: 2% NaOH at 60 °C for 2 h and the ozone initial pH 9 for 35 min. D-9-35: 2% NaOH at 80 °C for 4 h and the ozone initial pH 9 for 35 min. Blank: untreated degreased stover.

Comparing the spectra in Figure 5, the peak was obvious at 113 ppm in untreated stover. In A-5-25 and A-9-25, the peak at 106–153 ppm area was obvious, the peak in A-5-25 was mainly at 124 ppm, the peak in A-9-25 shifted to 134.2 ppm. It showed that with the initial pH of co-pretreatment changed from 5 to 9, the aromatic ether bond fragmented and free phenolic hydroxyl group formed. But the peak of disappeared in B-9-25 and the peak was not present in all ozone treatments for 35 min, it presumed that the corresponding aromatic lignin in this area was sensitive to the ozone initial pH 9 for 35 min and 4% NaOH. The untreated stover and A-5-25 treatment group had significant levels of aliphatic hydroxy lignin in the 20–35 ppm area but disappeared after ozone treatment at pH 9, indicating that the corresponding aromatic substances in this region were more sensitive to the ozone initial pH 9.

## 4. Conclusions

The optimal pretreatment condition was found to be 2% (*w*/*w*) NaOH treated at 80 °C for 2 h followed by ozone treatment for 25 min with an initial pH 9 and the maximum efficiency (91.73%) of enzymatic hydrolysis of cellulose was achieved. The promoting effect of three components in corn stover on the cellulose enzymatic hydrolysis was different with pretreatment conditions. Under the optimum pretreatment condition, the relative content of cellulose in the treated stover was 62.48%, the removal rate of lignin was 84.35% and the relative content of hemicellulose was 13.74%.

The results of SEM observation showed that the intensive structure of stover fiber changed to different degrees after synergistic treatment, many pores appeared on the surface and the fiber bundle was exposed. All of these increased the substrate accessibility of enzyme. The XRD characterization of the cellulose crystalline state showed that the synergistic pretreatment could change the crystal structure and crystallographic index, expand the interlayer spacing and so that the crystalline state of cellulose was more conducive to enzymatic hydrolysis. The FTIR characterization of chemical bond properties of stover before and after pretreatment showed that the pretreatment could effectively break the hemicellulose bond, the linkage bond between lignin and other carbohydrate and the intra-/inter-molecular hydrogen bond between the cellulose and other carbohydrate. The CP/MAS ^13^C-NMR determination of different positions of carbon in stover showed that pretreatment was beneficial to the removal of acetyl groups in hemicellulose and -OCH_3_ in lignin. All changes were conducive to the promotion of cellulose enzymatic hydrolysis. Finally, it emphasized that in this paper, corn stover was a representative of lignocellulose and the pretreatment method used in this article was suitable for other lignocellulosic materials [24].

## Figures and Tables

**Table 1:** Level of NaOH pretreatment factors.

Factor	NaOH Pretreatment Conditions		
NaOH Pretreatment Temperature	40 °C	60 °C	80 °C
NaOH Pretreatment Time	2 h	3 h	4 h

Note: In order to facilitate the description of the structure of typical pre-treated samples, a special nomenclature for NaOH pre-treatment conditions were as follows: A: 2% NaOH at 80 °C for 2 h; B: 2% NaOH at 80 °C for 3 h; C: 2% NaOH at 60 °C for 2 h; D: 2% NaOH at 80 °C for 4 h.

**Table 2:** Level of Ozone Pretreatment factors.

Factor	Ozone Pretreatment Conditions	
Ozone treatment Initial pH	5	9
Ozone treatment time	25 min	35 min

**Table 3:** Assignments of characteristic absorption of samples.

Number	Wavelength/cm^−1^	Absorption Band Attribution
1	898	Vibration of β-glycosidic bonds in cellulose and hemicellulose
2	1051	The bending of hydroxyl groups in lignin
3	1250	Ether bond between lignin and carbohydrates (β-*O*-4)
4	1370	Phenolic hydroxyl groups in lignin
5	1427	Methoxy in lignin (–OCH_3_)
6	1454	Methoxy in lignin (–OCH_3_)
7	1515	Extension of C=C on Lignin Aromatic Rings
8	1605	Lignin aromatic skeleton vibration
9	1654	Conjugated carbonyls in lignin
10	1704	Non-conjugated carbonyls in lignin degradation products
11	1732	Ether bond between lignin and carbohydrate (non-conjugated ketone and carboxyl group C=O stretch)

## Supplementary Materials

This study optimized conditions of sodium hydroxide synergistic ozone pretreatment. Preconditioning conditions of this experiment were determined based on a large number of previous experiments in our laboratory. The most representative is the analysis of surface area and porosity. The following is available in supplementary materials. Figure S1 showed the specific surface area changes with the increase of alkali treatment temperature. Figure S2: the specific surface area of corn stover changed with the NaOH treatment time increases. Figure S3 was about the particle size changes in the different pH value of ozone treatment in NaOH combined with ozone treatment. Figure S4: the effect of ozone treatment time on the specific surface area of corn stover was carried out. The mechanism of ozonation is revealed clearly by the analysis of specific surface area.

## Nomenclature

AT	single NaOH pretreatment
OT	single ozone pretreatment
AT-OT	the combine pretreatment with sodium hydroxide and ozone
G	guaiacyllignin
S	syringyllignin
