# Supplementary material for: Enhanced Enzymatic Hydrolysis and Structural Features of Corn Stover by NaOH and Ozone Combined Pretreatment
Source: Molecules. 2018 May 29;23(6):1300. doi: 10.3390/molecules23061300 (PMC6100291; doi:10.3390/molecules23061300)
Supplement: Supplementary file 1 [file molecules-23-01300-s001.pdf]

Supplementary Materials

1. Resultsand Discussion of surface area and porosity.

As shown in Figuer S1, the specific surface area decreases with the increase of temperature. When the temperature of alkali treatment increases from 40 ℃ to 60 ℃, the specific surface area of stover decreases by only 7.2 um , the specific surface area of straw decreased by 36.7 um when the temperature is raised to 100 ℃, the specific surface area of stover is reduced by 31.1% and the surface area is reduced to 108.3 um in diameter when treated by NaOH and ozone. While the specific surface area ratio of the NaOH combined with ozone treatment and the NaOH-treated stover alone at 80 ℃ to 100 ℃, the difference between the diameters increase from 1.9 um at 40 ℃ to 14.2 um.

As can be seen from Figure S2, as the NaOH treatment time increases, the specific surface area of corn stover showed a slow decline in the diameter. When the NaOH treatment time was 2 h, the specific surface area of NaOHcombined with ozone treatment decreased by 11.2 um compared with that of NaOH treatment alone.

As shown in Figure S3, when the pH value of ozone treatment in NaOHcombined with ozone treatment gradually decreases, the particle size of stover decreases gradually. The specific surface area of stover is 164.08 um when the pH value is 11, and the specific surface area of stover decreased rapidly to 138.01 um and the specific surface area decreased by 26.07 um, when the pH value of the solution decreased to 9.

As can be seen from Figure S4, when the ozone treatment time increases, the specific surface area of stover decreases slightly first, then rapidly decreases and finally slowly decreases. When the treatment time was 10 min, the specific surface area of stover decreases by 4.68 um compares with 5 min. When the treatment time increases from 10 min to 30 min, the specific surface area of stover decreases rapidly to 99.69 um The reason for this drastic decrease may be that ozone treatment degrades the lignin in the shallow layer of strover particles and causes the decrease of specific surface area diameter. However, the specific surface area diameter of stover decreases slowly within 30 min to 110 min, and the reason for the slowing down may be that the denser structure of corn stover hinders the penetration of ozone into the interior.

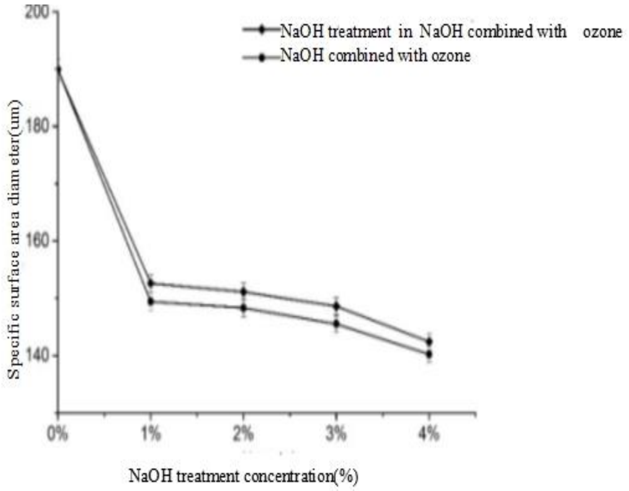

Figure S1. Effect of NaOH concentration on specific.

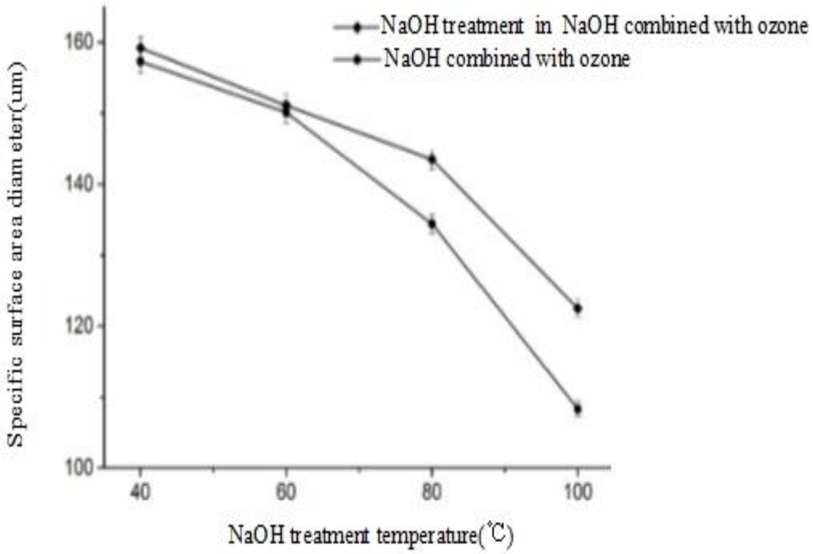

Figure S2. Effect of NaOH temperature on specific surfacearea diameter of corn stoversurface area diameter of corn stover.

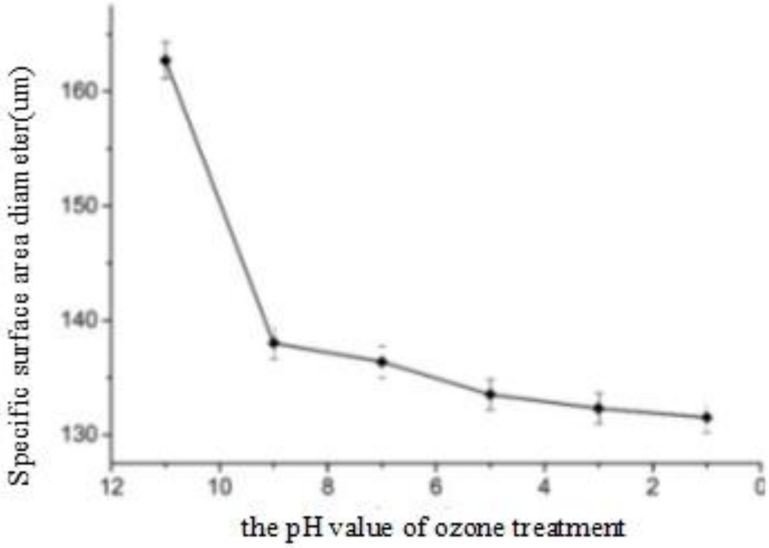

Figure S3. Effect of ozone pretreatment pH on specific.

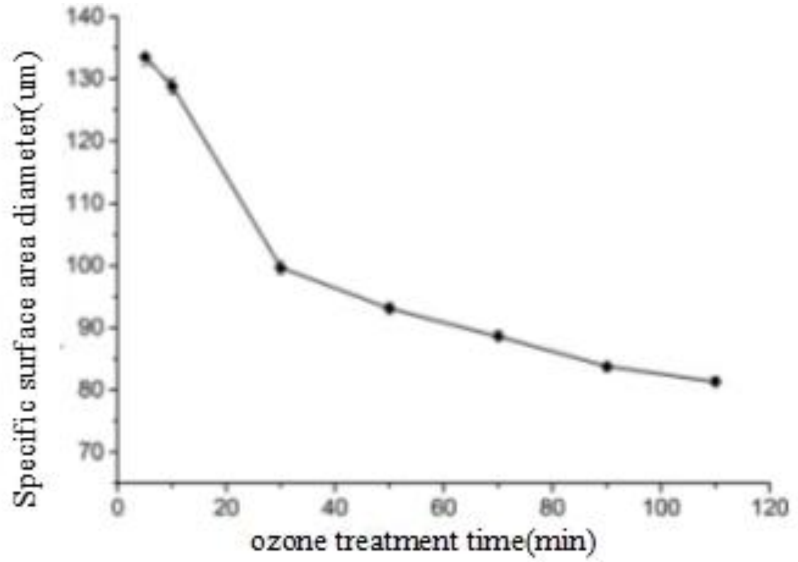

Figure S4. Effect of oxidation time ofozone on specific surface area diameter of corn stoversurface area diameter of corn stover.

2. The mechanism of ozonation

Ozone treatment can oxidatively break the structure of bound cellulose, rapidly oxidize and degrade lignin composed of phenylpropane structural units, accelerate the dissolution of hemicellulose, destroy the lignocellulosic network structure, and fully expose the cellulose. Its oxidative degradation mainly through the molecular structure of high molecular weight lignin to a small molecular weight of the basic structural unit of benzene, benzene to benzoic acid, and then be open-loop oxidation of carboxylic acids and aldehydes, and finally be oxidized to acetic acid, carbon dioxide and water. The research in this article is based on a large number of previous studies on the mechanism of NaOH combined ozone treatment.
